# Trends in Multiplex Immunoassays for In Vitro Diagnostics and Point-of-Care Testing

**DOI:** 10.3390/diagnostics11091630

**Published:** 2021-09-07

**Authors:** Sandeep Kumar Vashist

**Affiliations:** Sensing Self Pte. Ltd., 160 Robinson Road, #20-03, Singapore Business Federation Ctr., Singapore 068914, Singapore; dr.sandeep@sensingself.me

The tremendous advances in multiplex immunoassays (MIAs) are leading to novel in vitro diagnostics (IVD) and point-of-care testing (POCT). MIAs can simultaneously detect numerous analytes in a single sample, which facilitates the diagnosis of many complex diseases. Various clinical score-based diagnostic algorithms have already been developed for several complex diseases, where the clinical score is determined by assigning appropriate weightage to various biomarkers based on their contribution to the disease. Despite a wide range of MIA formats being developed, only a few have been commercialized. There is a need for considerable improvements in MIAs so that they are analytically superior and can compete with the most extensively used automated IAs. The readout of most MIAs is still completed by bulky and expensive reader devices, which emphasizes the need for compact, handheld multiplex readers. Further, the clinical utility, reimbursement models, pathophysiological range of analytes, nature and dilution of samples, and the reagents used to develop an MIA need to be analyzed stringently. This manuscript provides guided insights into MIA formats and discusses the challenges and future directions.

A wide range of MIA formats has been demonstrated during the last decade. The need for multiplex analyte detection has also been substantiated as it could critically improve diagnosis, monitoring, and management of complex diseases. MIAs would lead to better health outcomes by enabling early and accurate diagnosis of complex diseases. It would provide the desired opportunity to healthcare professionals as they could start the treatment of patients at the earliest possible time, thereby preventing costly late-stage complications and mortality. Rapid clinical diagnosis and prompt treatment are critical in intensive care units and emergencies, where immediate clinical decisions must be taken. Further, complex diseases, such as sepsis, require quantitative analysis of many biomarkers and an advanced clinical scoring algorithm. The recent advances in MIAs have demonstrated tremendous utility for clinical IVD and POCT [1], although they would also be useful for veterinary sciences, food safety, and environmental testing [2].

Most MIA formats could perform multiplex detection of a limited number of analytes and require expensive and bulky benchtop readers. Therefore, there is a need to develop rapid and cost-effective POC MIAs and portable handheld multiplex readers, which could be used at any place and time by users who have basic operator skills. In addition, the emerging trend towards smart digital healthcare, where the diagnostic devices are equipped with mobile healthcare and other smart features, would pave the way to personalized healthcare monitoring and management.

A prospective MIA format uses spot microarray on a solid substrate, which can simultaneously detect several analytes in a sample. It has been used by Scienion, R-Biopharma, Biovendor, TestLine, and some other companies. The preparation of microarray spots on a substrate requires complex preparation and expensive microarray spotters with precise control of spotted volume in nL-pL. However, most spot-microarray-based MIAs employ a conventional manual ELISA procedure that takes a lot of time and involves multiple steps. Further, there is a significant risk of misinterpretation due to the non-specific background signal when nitrocellulose or nylon membranes are used. The readout of spot microarrays is completed by expensive and bulky readers using an advanced imaging algorithm. The signal readout is mainly optical via colorimetry, fluorescence [3], or chemiluminescence [4] that involves image capture using a scanning charge-coupled device (CCD) or complementary metal-oxide-semiconductor (CMOS) camera. It is important to ensure a good morphology of spots and maintain an appropriate distance between them as these variables could critically impact the performance of MIAs. There is a possibility of crosstalk between different IAs as the assay components from a particular spot can diffuse to adjacent spots [5,6,7]. Additionally, most such MIAs are only qualitative that do not address the clinical need for quantitative results. Moreover, there is a need for a fully automated MIA where all the assay components of the MIA are integrated into a plug-and-play cartridge and all operations are performed by a benchtop analyzer, thereby obviating any manual handling by the users. However, it should be noted that it is not always possible to multiplex all analytes into the same well of 96-well microtiter plate (MTP) as the sample dilutions required for various analytes are very different. Moreover, it is possible that the assay formats for different analytes may also vary as competitive immunoassay (IA) is used for small analytes while sandwich IA is employed for big analytes. Therefore, spot microarray-based MIA format has limited applications in IVD and as most such MIAs have a total IA duration of more than an hour, they are not ideal for POCT. 

Another MIA format involves the electrochemical detection of multiple analytes using a microelectrode array. An example is the ElectraSense platform from Custom Array Inc., USA, which detects multiple analytes on a CMOS-based chip with platinum microelectrodes via electrochemical detection. A handheld reader measures the signal in less than a minute [8,9]. The chip can be reused up to four times, which might be useful for research but inappropriate for clinical diagnostics. The main limitation is that it is expensive and involves complex fabrication procedures, making it inappropriate for clinical IVD applications.

The xMAP^®^ technology by Luminex Corp., USA [10], widely used to develop bead-based MIAs [11,12,13], can detect up to 100 analytes in a single well of 96-well MTP using numerous distinctly colored bead sets. The xMAP^®^-based MIA detects the analytes in a sample by binding to capture antibody (Ab)-bound color-coded micron-sized polystyrene beads known as microspheres, followed by subsequent detection via binding to biotinylated detection Ab and streptavidin-labeled fluorescent dye (Figure 1). The readout is performed by an analyzer comprising multiple lasers or LEDs and high-speed digital-signal processors. Luminex has developed two flow cytometry-based analyzers, i.e., Luminex^®^ 100/200™. A laser or LED identifies the specific microsphere set by exciting the microsphere’s internal dyes, while a second laser or LED excites the fluorescent dye bound to the detection Ab. The high-speed digital signal processors enable quantitative analysis of multiple analytes by measuring the fluorescent signals from each microsphere. There is a possibility to employ magnetic beads that might be of interest to many IVD companies to develop automated IAs. However, the limitations of this format are the need for expensive instruments and prolonged IA duration.

The high sensitivity electrochemiluminescent ELISA [14] by Meso Scale Diagnostics LLC is another prospective MIA that detects multiple analytes in a sample using carbon electrode surface-based microwell plates and a wash-free sandwich IA. It employs SULFO-TAG-labeled detection Ab that emits light upon electrochemical stimulation (Figure 2). The format uses a simple procedure obviating the labor-intensive washing steps and has good analytical performance comparable to Luminex *x*MAP^®^ MIA [15,16,17]. 

Lateral flow IA (LFIA) are the most simple, rapid, and cost-effective IA formats for POCT at homes, remote settings, decentralized laboratories, and point-of-need settings. The tremendous utility and potential of such assays have already been witnessed by billions of coronavirus disease 2019 (COVID-19) antigen tests that have been used worldwide during the current pandemic. Apart from the professional IVD use as COVID-19 antigen tests, they have also been approved for self-use. They are available in singleplex as well as multiplex format. For example, Sensing Self Pte. Ltd., Singapore developed a COVID-19 rapid antigen test and a multiplex rapid antigen test for COVID-19/MERS-CoV/Influenza A/B [19] (Figure 3). Several multiplex LFIA tests for other analytes have already been developed [20], which mainly employ the detection of the optical signal [21,22,23,24], although a few use electrochemical detection [25,26]. A prospective quantitative LFIA-based MIA has been developed by Euroimmun (a PerkinElmer company), Germany. It employs EUROLINE membrane test strips [27] and a flatbed scanner and imaging system based EUROLineScan [28] for the diagnosis of autoimmune liver diseases, anti-nuclear antibody (ANA), myositis, T.O.R.C.H, extractable nuclear antigens (ENA), and other diseases. Another innovative quantitative LFIA-based MIA is from Quidel Corporation, USA, which uses the Triage platform and fluorescence-detection-based LFIAs to detect cardiac biomarkers and drugs in whole blood, plasma, or urine [29,30,31] in less than 20 min. A portable fluorometer, Triage^®^ MeterPro [31], is used to obtain rapid POCT results. Several pocket-sized smart LFIA readers, equipped with mobile healthcare tools and Cloud computing, have also been developed by different groups for quantitative LFIAs, which could increase the outreach of POCT and healthcare. Some prominent examples are the smart LFIA readers developed by Cellmic, USA and BBI Solutions, UK.

Microfluidic paper-based analytical devices (MF-PADs) have been used for MIAs due to their ability to manipulate liquids at a high level and adaptation of various microfluidic operations (such as mixing, splitting, separation, and filtration). They are ideal for developing cost-effective diagnostics for developing nations, but they have not been a commercial success due to the increased efforts required for mass production, concerns about their reproducibility, and the need for a simplified operational procedure. Although the conventional MF-PAD-based MIA employs colorimetric detection via naked eyes and are thus qualitative or semiquantitative, the development of smart readers could lead to quantitative MF-PAD-based MIAs. Of interest is the MF-PAD-based colorimetric MIA for on-site liver function testing by determining the levels of aspartate aminotransferase (AST) and alanine aminotransferase (ALT) in whole blood in less than 15 min [32]. The fabrication and MIA procedure are illustrated in Figure 4. Another prospective MIA format is the electrochemiluminescent MF-PAD that detects four tumor markers in human plasma [33].

Microfluidics (MF)-based MIAs for the quantitative detection of analytes have been demonstrated [35]. They require an optimal MF array and design, and the manipulation of fluids by a number of pneumatic valves integrated into polydimethylsiloxane (PDMS)-based devices [36,37,38]. A prospective MF-based MIA, comprising of a disposable MF cartridge with preloaded reagents and a handheld analyzer [39], was used for the detection of HIV antigen, syphilis antigen, BSA and Ab to goat IgG by employing four detection sites in series in each MF channel. The optical density signal, obtained by reducing silver ions on detection Ab-tagged gold nanoparticles (AuNPs), was measured using a low-cost and compact reader comprising LEDs and photodetectors. The MIA detects the analytes in less than 20 min using only 1 mL of finger-pricked whole blood. The results correlated well with those obtained by an established reference test. However, the major limitation of MF-based MIA formats is that they are very limited in multiplexing capabilities as they could only detect a few analytes. Moreover, it is difficult to make the MF operations flexible and open for the various IA formats.

An innovative MIA format, using a centrifugal MF (CMF) based lab-on-a-disc platform, involves performing various IA steps via MF operations that navigate the fluids through the microchannels using centrifugal forces. Gyros Protein Technologies AB, Sweden, Abaxis, Inc., USA, and Samsung, South Korea, have developed automated MIAs performed on a benchtop analyzer. The optical signal from each IA is read in the analyzer by an integrated reader. This MIA format has low sample requirement, rapid sample-to-answer time, and automated operation. Although this automated MIA format is close to the fully automated random-access analyzer based chemiluminescent immunoassays that are widely used in clinical labs, there are still critical improvements required in terms of multiplexing, robustness, and IA procedures. The POC Piccolo Xpress™ whole blood chemistry analyzer [40] (Figure 5A) by Abaxis Inc., USA can perform up to 14 different tests on a single barcoded LabDisk that contains all prestored reagents. Similarly, Gyros Protein Technologies, Sweden has developed the Gyrolab instrument (Gyrolab xPlore™ or Gyrolab™ xP workstation) [41] and Gyrolab Bioaffy CDs [42] (Figure 5B). While Gyrolab xPlore™ performs a single IA on a Gyrolab Bioaffy CD, the Gyrolab™ xP workstation performs multiple IAs by running up to five Gyrolab Bioaffy CDs. Samsung LABGEO IB10 [43], the handheld POC analyzer from Samsung, South Korea, employs CMF-based automated MIAs that detects multiple analytes in less than 20 min. It could detect up to three analytes in a single run and has smart mobile healthcare tools. Samsung has developed IAs for troponin I, myoglobin, CK-MB, thyroid stimulating hormone, procalcitonin, and other analytes. The lab-on-a-disc, containing all the prestored reagents for the MIAs, is stable at room temperature for a month.

Quanterix, USA has developed an innovative automated MIA [45] based on a highly sensitive Simoa^®^ Planar Array technology [46,47,48]. It can measure up to 10-plex biomarkers. The 96-well MTP are spotted with Ab via a proprietary, high-precision, digital nanofluidic deposition. This is followed by the addition of diluted samples and standards to each MTP well to create a unique surface chemistry and vortex effect. Subsequently, the biotinylated detection Ab is added that creates sandwich immune complexes. Thereafter, a high sensitivity HRP-labeled streptavidin and a chemiluminescent substrate are added in consecutive steps. Finally, an ultrasensitive CCD camera images the MTP wells and provides the signal intensity produced by each spot. The company provides Simoa^®^ SP-X, a benchtop multiplex biomarker detection system, which images a 96-well MTP in less than 2.5 min without any requirement of calibration. It also provides a fully automated HD-X Analyzer™ that can measure up to six biomarkers in a single assay at fg/mL concentrations. 

The MIAs would be useful for the clinical diagnosis of many complex diseases as they involve the detection of many biomarkers. The use of advanced diagnostic algorithm based on a clinical score, which is determined by assigning specific weightage to each biomarker in the multiplex panel, would further improve the diagnosis and lead to differentiation of diseases. However, despite several MIAs developed by companies such as Luminex Corp., Meso Scale Diagnostics LLC, Gyrolab, Abaxis, R-biopharm, Biovendor, Testline, and Scienion, the market penetration of MIAs is still negligible in comparison to automated IAs that occupy a predominant market share. This is mainly due to the limitations and pending concerns of MIAs. However, the continuous developments in microfluidic technologies, POC platforms, novel biosensors, lab-on-a-chip, new wash-free IA formats, and smart technologies are leading to critically improved MIAs in the coming years. The cost-effectiveness, simplicity, robustness, analytical performance, ease of manufacture, and clinical utility will play a key role in the market acceptance and penetration of MIAs.

It is critical that the bioanalytical performances of IAs for various biomarkers in a MIA should correlate well with the results obtained from established predicate IAs for each biomarker. If a single biomarker in the MIA format does not meet the desired performance, the whole MIA would fail in terms of development. Therefore, the development of MIA carries a high risk and is very costly in comparison to automated IAs that detect a single biomarker. On the other hand, there are several limitations, which makes it very challenging for MIA formats to penetrate the market. One of the most predominant concerns is the reimbursement of MIAs. If the physicians prescribe only IA for the determination of selected biomarkers instead of all the biomarkers in the MIA, the healthcare authorities cannot justify charging the costs of an entire MIA as this will not be approved by the reimbursement agencies. Therefore, the MIA companies need to conduct a thorough due diligence and discuss it with the healthcare authorities and reimbursement agencies before they start developing a particular MIA. In some cases, it is better to go for different combinations of biomarkers in separate kits so that the clinical needs are addressed, as demonstrated for COVID-19 testing by Sensing Self Pte. Ltd., Singapore [19].

Presently, LFIA based rapid multiplex test is the most prospective MIA format based on its rapid sample to provide a response in less than 20 min, and its ease of use, stability, and cost-effectiveness. It can be used both for professional IVD use and self-use, as evident during the current COVID-19 pandemic. However, there is an emerging trend towards POC, PADs, and MF-based MIAs. The MIA is still in its nascent stages, but it has tremendous utility for the clinical diagnosis of complex diseases. The continuous advances in complementary technologies and IA formats would lead to the development of critically improved MIAs in the coming years, which, in turn, would lead to better health outcomes.

## Figures and Tables

**Figure 1:**
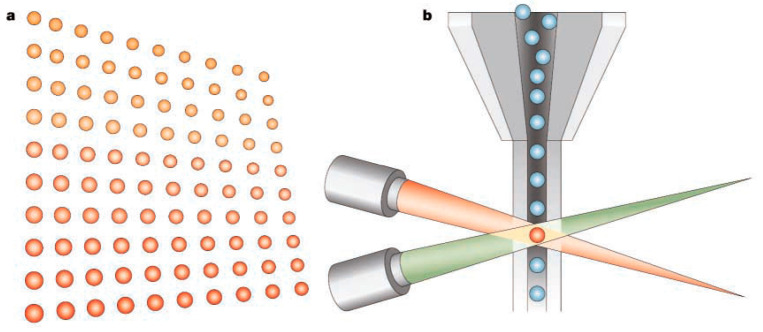
Luminex *x*MAP^®^ MIA format. (**a**) Polystyrene beads are internally colored with two different fluorescent dyes: red and infrared, with up to 100 distinct bead regions generated by using different concentrations of red and infrared dyes. Each bead region, bound to a different capture Ab, detects its specific analyte, followed by the binding of a biotinylated detection Ab and streptavidin-conjugated phycoerythrin (reporter dye). (**b**) The beads are identified individually in a rapidly flowing fluid stream that passes by two laser beams: red classification laser (635 nm) or LED reveals the color code of the bead region, and green reporter laser (532 nm) or LED determines the analyte concentration by measuring the reporter fluorescence intensity [13]. Reproduced with permission from Elsevier B.V. [13].

**Figure 2:**
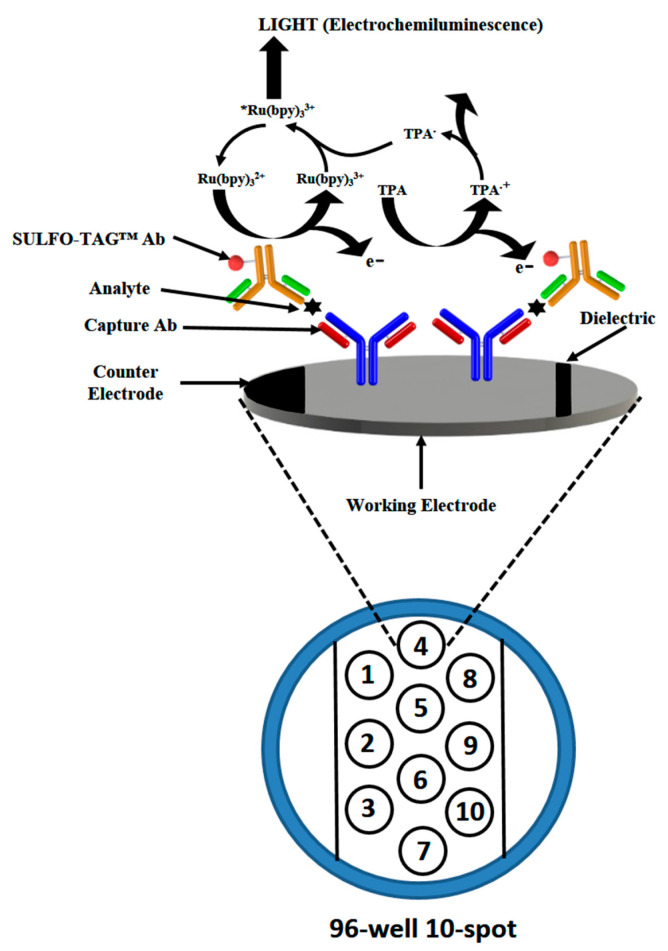
Wash-free electrochemiluminescent ELISA from Meso Scale Diagnostics LLC. The electrochemical stimulation leads to light emission from the carbon electrode surface-based microwell plates due to specific SULFO-TAG™ labels bound to the detection Ab. Reproduced with permission from Elsevier B.V. [18].

**Figure 3:**
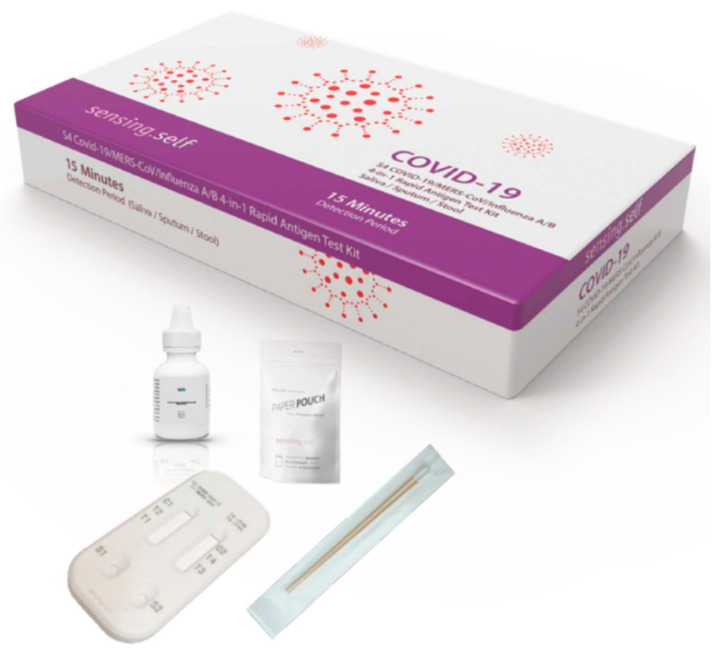
A multiplex rapid antigen test for COVID-19/MERS-CoV/Influenza A/B, developed by Sensing Self Pte. Ltd., Singapore.

**Figure 4:**
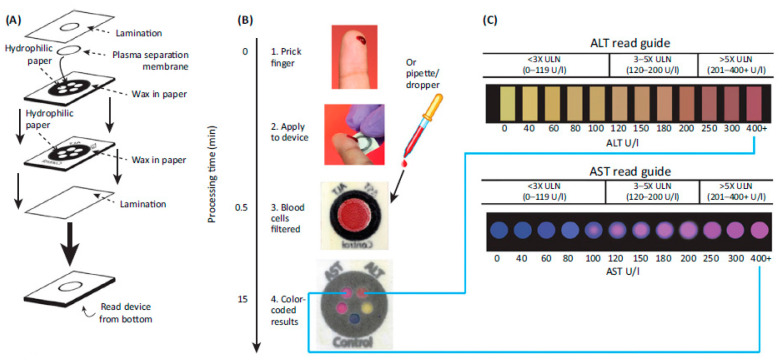
MIA for on-site liver function testing using the MF-PAD. (**A**) Fabrication procedure for MF-PAD. (**B**) IA procedure. (**C**) Colorimetric readout guides for the quantitative determination of liver function enzymes. Reproduced with permission from AAAS [34].

**Figure 5:**
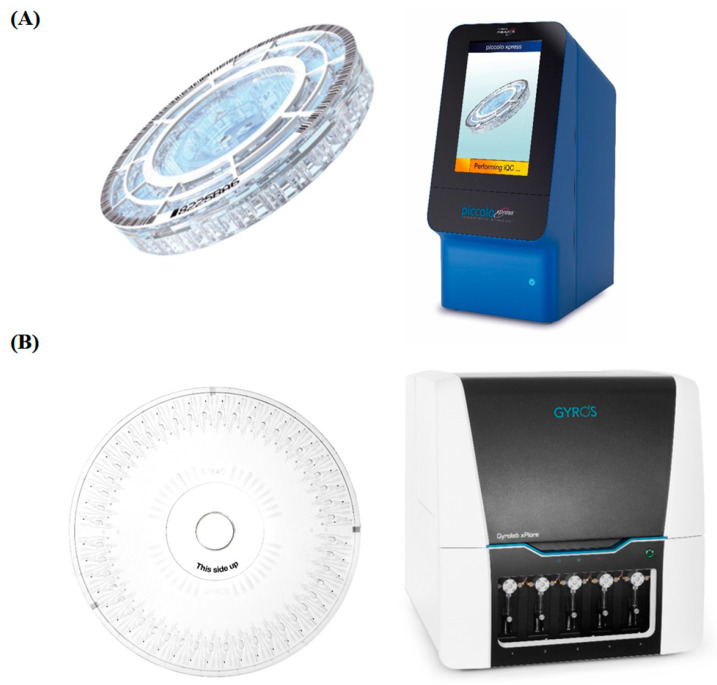
(**A**) (Left) Centrifugal microfluidics platform, i.e., LabDisk, for multiplex detection; and, (right) Piccolo Xpress chemistry analyzer for fully automated IA. (**B**) (Left) LabDisk platform for fully automated IA; and, (right) Gyrolab Xplore™ system enabling fully automated IA. Reproduced with permission from Elsevier B.V. [18,44].
